# Ethnic variations in overweight and obesity among children over time: findings from analyses of the Health Surveys for England 1998–2009

**DOI:** 10.1111/j.2047-6310.2013.00159.x

**Published:** 2013-04-02

**Authors:** S Karlsen, S Morris, S Kinra, L Vallejo-Torres, R M Viner

**Affiliations:** 1 Department of Applied Health Research, University College LondonLondon, UK; 2 Department of Non-Communicable Disease Epidemiology, London School of Hygiene and Tropical MedicineLondon, UK; 3 Clinical Trials Unit, University College LondonLondon, UK; 4 Institute of Child Health, University College LondonLondon, UK

**Keywords:** Child health, ethnicity, obesity, overweight

## Abstract

**Background:**

The increase in the prevalence of obesity among children and adolescents in England since the mid-1990s has been dramatic. Cross-sectional evidence suggests ethnic variations in childhood obesity prevalence.

**Objectives:**

The objective of the study was to examine whether and how ethnic variations in childhood overweight/obesity have changed over time, and are affected by socioeconomic factors.

**Method:**

This study uses logistic regression to analyse ethnic differences in the relative likelihood of being at or above the age- and gender-specific thresholds for overweight and obesity developed by the International Obesity Task Force among children aged between 2 and 15 from 11 ethnic groups included in the Health Surveys for England between 1998 and 2009, adjusting for age, gender, year of data collection and equivalized household income. We separately analyse the likelihood of being at or above the thresholds for overweight (but below those for obesity) and obesity.

**Results:**

Trends in overweight/obesity over time among ethnic minority groups do not follow those of white English children. Black African children had higher rates of overweight and obesity, which appear to have peaked, and black Caribbean children had higher rates of obesity than other groups examined, which appear to continue rising. These differences were not explained by socioeconomic variations between groups.

**Conclusion:**

Policies are required that encourage healthy lifestyles among ethnic minority young people, while engaging with the complexities associated with these choices during childhood and adolescence.

## What is already known about this subject

- The increase in the prevalence of obesity among children and adolescents in England has stabilized following a peak in 2004 and 2005.
- This stabilization conceals continued rises among more deprived and certain ethnic minority groups.
- It is unclear which ethnic groups in the UK experience the highest rates of overweight/obesity, how this has changed over time and how these patterns relate to socioeconomic differences between the groups.

## What this study adds

- Trends over time suggested that overweight/obesity rates for ethnic minority groups had not peaked, unlike those for white English groups.
- Black African children had higher rates of overweight and obesity, and black Caribbean children had higher rates of obesity.
- Differences were not explained by variations in equivalized household income.

## Introduction

The increase in the prevalence of obesity among children and adolescents in England since the mid-1990s has been dramatic. Findings for children aged between 2 and 15 included in the Health Surveys for England (HSE) indicate that the prevalence of obesity increased by five percentage points among boys (from 11 to 16%) and three percentage points among girls (from 12 to 15%) between 1995 and 2009 1. Childhood obesity is associated with a number of long-term and immediate-health risks and impacts on quality of life 2–4. Identifying opportunities to address these trends, therefore, remains an important focus for government initiatives and health service action 5.

Several studies have identified an ethnic variation in levels of overweight and obesity among children. Yet, studies report different ethnic groups to be at highest risk, in part because of the varying approaches taken to assessing overweight/obesity and differences in the ethnic groups included in these analyses 6–9. Establishing these trends more definitively is important as although there is evidence that obesity rates have stabilized more recently (following a peak between 2004 and 2005) 1,10,11, stabilization appears to be concentrated among white groups with continued rises among more deprived and certain ethnic minority groups 11.

This paper examines the extent of ethnic variations in overweight and obesity among those aged between 2 and 15 years and how these have changed over time, using data from the HSE 1998–2009 12–23. The study extends earlier work by exploring ethnic variations in overweight/obesity over time (during a period of dramatic increases in childhood and adolescent body mass index [BMI] followed by a period of stabilization for some groups) and by examining variations between more disaggregated ethnic groupings than have been previously studied. The latter is feasible as a consequence of the particular sampling approaches adopted by the HSE and the additional power offered by the pooling of data over a 12-year period. It takes advantage of the opportunities offered by the HSE to provide an assessment of variations in overweight and obesity between ethnic groups within 4-year periods and between periods within ethnic groups. Overweight and obesity are defined using International Obesity Task Force (IOTF) definitions 24. Data are also pooled across the 12-year period for each ethnic group and stratified by gender, controlling for the effects of year of data collection, following evidence that recent trends in obesity have varied between (white) boys and girls 1.

Obesity in adolescence and young adulthood has been shown to have a negative effect on educational attainment and income in young adulthood 2. There is empirical evidence of a negative association between childhood obesity and equivalized household income 10,25,26 and other measures of household 27 and area 8,10 socioeconomic classification in the UK. People with ethnic minority backgrounds often experience socioeconomic disadvantage relative to the ethnic majority in the UK 28,29. Although social class differences have not been found to explain ethnic variations in overweight and obesity in earlier studies 6,7, we explore the impact of ethnic variations in income as the additional sample power offered here may provide further insight into the drivers of any ethnic variations in childhood obesity than that offered previously.

## Data and methods

### Data

The HSE are designed to provide nationally and regionally representative data about the population aged two and over living in private households in England. A multi-stage stratified probability sampling design is employed based on postcode sectors, where areas, then addresses and then individuals within addresses are identified to be included in the study.

At each address, all households, and all persons in them, were eligible for inclusion in the survey. The exception was households containing three or more children aged 0–15, where two were selected at random. The 1999 13 and 2004 18 surveys oversampled people with black Caribbean, black African, Indian, Pakistani, Bangladeshi, Irish or Chinese heritage. The 1999 and 2004 surveys did not include representative samples of white people. The comparison white sample used in analysis of the 1999 and 2004 surveys is taken from the preceding survey 13,18. These analyses, therefore, combine the 1998 and 1999 surveys and the 2003 and 2004 surveys for analyses of single years.

Children aged 13–15 were interviewed directly, once permission had been received from the child's parent or guardian. Information on younger children was obtained from a parent, although whenever possible younger children were present while the parent answered questions about their health, which ensured that the child could contribute information where possible.

### Variables

Eleven ethnic categories were included in these analyses (see Tables; associated sample sizes are in Tables 3 and 4). Ethnicity was categorized using responses to a question ‘To which of these groups do you consider you belong?’ Answer options included ‘white’, ‘Mixed ethnic group’, ‘black or black British’, ‘Asian or Asian British’ and ‘any other group’. Respondents were then asked to further clarify their ethnic background in response to the questions ‘Do you have family origins which are. …’ and ‘What is your cultural background?’. Nominated response options included black Caribbean, black African, Indian, Pakistani, Indian Caribbean, African–Indian, Chinese, Japanese, Filipino and Vietnamese. Responses also included a range of mixed ethnicities, which were a combination of white and the ethnic minority backgrounds described earlier. Respondents responding ‘white’ were also asked ‘Were you or either of your parents born in Ireland?’ Responses have a strong correlation with Census ethnic identity categories 28. Those reporting a ‘mixed’ ethnicity were allocated to the relevant ethnic minority group for these analyses.

Overweight and obesity were defined using BMI, calculated by dividing a child's weight (in kilogrammes [kg] ) by the square of their height (in metres [m] ). In the HSE, height and weight are measured either during a nurse visit or by the interviewer and are not self-reported, reducing the likelihood of measurement error. Height is measured without shoes or socks using a portable stadiometer and a Frankfort Plane Card. Weight measurements are taken without shoes or other heavy garments/items using Soehnle (Soehnel, Nassau/Lahn, Germany), Seca (Seca, Hamburg, Germany) or Tanita (Tanita, Illinois, USA) electronic bathroom scales, calibrated for the Health Survey. BMI values were converted into underweight, normal weight, overweight and obese categories using cut-offs identified by the IOTF 24. These are specified by age in 6-month categories (from 2 to 18 years) and gender and are calculated to be equivalent to adult BMI of over 30 kg m^−2^ at age 18. They were obtained by pooling data from Brazil, Great Britain, Hong Kong, the Netherlands, Singapore and the USA 24. We examined separately ethnic differences in the proportions of children at or above the IOTF thresholds for overweight (but below those for obesity) and those at or above the IOTF thresholds for obesity.

The following variables were included as likely confounding factors: age (measured continuously and included as a quadratic function); gender; year; interactions between ethnicity and year; and annual household income, equivalized to account for household composition using equivalence scales reported in the HSE.

### Analyses

Regression analysis was used to estimate the impact of ethnic differences in the probability of children being overweight (but not obese) and obese, and to explore trends in overweight (but not obesity) and obesity by ethnic groups over time. We examined models that combined all the years of data that were available (‘combined data’) and also models that are stratified by time period (‘stratified data’).

#### Analyses using combined data

We first consider models using data for all years combined (1998–2009) to explore ethnic variations in overweight and obesity prevalence. Logistic regression models were used to separately regress overweight (but not obesity) and obesity (1 = overweight, obese, 0 = normal or underweight) against ethnic group with white English as the omitted category, controlling for age and gender, and year of data. Models were run separately for each gender and with and without controlling for equivalized household income. The results from the models were used to calculate predictive margins, i.e., adjusted probabilities of overweight and obesity in each ethnic group, fixing the other variables at their sample mean values; we report point estimates and 95% confidence limits.

Ethnic differences were examined in every model by testing the joint significance of the ethnic group variables using Wald tests. Differences between specific ethnic groups can be identified by comparing the 95% confidence intervals around the predicted prevalence. Variations reported as being ‘statistically significant’ correspond to a *P*-value of less than 0.05 and those reported as ‘approaching statistical significance’ or ‘indicative’ of significant variation correspond to a *P*-value of between 0.05 and 0.1.

#### Analyses using stratified data

Similar models were run stratified by year of data defined in three 4-year periods (1998–2001, 2002–2005 and 2006–2009). This allows us to explore trends in overweight and obesity between ethnic groups over time. The decision to combine years in this way was based on analyses, focussing on the white English (the largest) group, which suggested no statistically significant variation in rates of overweight and obesity in 1998/1999, 2000 and 2001 (overweight χ^2^ 3.10, *P*-value = 0.21; obesity χ^2^ 0.74, *P*-value = 0.69); 2002, 2003/2004 and 2005 (overweight χ^2^ 1.62, *P*-value = 0.44; obesity χ^2^ 0.16, *P*-value = 0.92); or in 2006, 2007, 2008 and 2009 (overweight χ^2^ 1.00, *P*-value = 0.80; obesity χ^2^ 0.54, *P*-value = 0.91). Combining years in this way provides additional sample power and avoids random fluctuations in BMI that might be seen in analyses based on single years, but do not reflect general trends. We also explored the significance of interactions between ethnicity categories and year of data and have included details of the change in single years as supplementary material.

Again, the joint significance of the ethnic group variables was tested in each model using Wald tests. We additionally tested for variations across years within each ethnic category.

In each model, weights were applied to correct for the unequal probabilities of selection for postcode sectors, addresses within postcode sectors and children within households. Data from the 1999 and 2004 surveys were also weighted to allow for the oversampling of ethnic minority groups in these years, back to an estimate of the population distribution in these years. The standard errors were adjusted to allow for clustered sampling at the primary sampling unit (PSU) level within the HSE. This accounts for the possibility that observations within particular PSUs were not independent.

## Results

### Analyses using combined data

The prevalence of overweight (but not obesity) and obesity among children by ethnicity, adjusted for age and gender differences between the groups, are shown in Tables 1 and 2 using the combined data. The test of the joint significance of the ethnic categories is statistically significant in every model adjusted for age and gender suggesting significant ethnic variations both in the prevalence of overweight and the prevalence of obesity. In these models, the significant ethnic variations predominantly reflected a higher risk of black African children being overweight and a higher risk of black Caribbean and black African children being obese. There were a number of other substantive differences across ethnic groups, but showing wider and overlapping confidence intervals. This might indicate that other ethnic variations may have become statistically significant with the inclusion of additional children in these groups.

**Table 1:** Ethnic differences in the age- and gender-adjusted probabilities of children aged between 2 and 15 being at or above the International Obesity Task Force thresholds for overweight (but below those for obesity)*, rather than normal or under-weight – *combined data across all years*

	Both genders (1)	Boys (1)	Girls (1)	Both genders (2)	Boys (2)	Girls (2)
White English	0.19 [0.18, 0.19]	0.17 [0.16, 0.18]	0.21 [0.20, 0.22]	0.19 [0.18, 0.19]	0.17 [0.16, 0.18]	0.21 [0.20, 0.22]
White Irish	0.17 [0.14, 0.20]	0.17 [0.12, 0.22]	0.17 [0.12, 0.22]	0.17 [0.14, 0.20]	0.17 [0.12, 0.22]	0.17 [0.13, 0.22]
Other White	0.19 [0.16, 0.22]	0.18 [0.14, 0.22]	0.19 [0.15, 0.24]	0.19 [0.16, 0.22]	0.18 [0.14, 0.22]	0.20 [0.15, 0.24]
Black Caribbean	0.19 [0.16, 0.21]	0.15 [0.12, 0.19]	0.22 [0.18, 0.26]	0.18 [0.16, 0.21]	0.15 [0.12, 0.19]	0.21 [0.17, 0.26]
Black African	0.24 [0.20, 0.28]	0.20 [0.15, 0.24]	0.28 [0.23, 0.34]	0.24 [0.20, 0.27]	0.20 [0.15, 0.24]	0.28 [0.22, 0.33]
Indian	0.19 [0.16, 0.22]	0.16 [0.13, 0.20]	0.21 [0.16, 0.25]	0.19 [0.15, 0.22]	0.16 [0.13, 0.20]	0.21 [0.16, 0.25]
Pakistani	0.19 [0.16, 0.22]	0.19 [0.15, 0.24]	0.19 [0.15, 0.23]	0.19 [0.15, 0.22]	0.19 [0.15, 0.24]	0.18 [0.14, 0.23]
Bangladeshi	0.17 [0.12, 0.22]	0.11 [0.07, 0.16]	0.23 [0.15, 0.31]	0.16 [0.12, 0.21]	0.11 [0.06, 0.16]	0.22 [0.14, 0.30]
Other South Asian	0.14 [0.11, 0.17]	0.12 [0.09, 0.16]	0.16 [0.12, 0.20]	0.14 [0.11, 0.17]	0.12 [0.09, 0.16]	0.16 [0.12, 0.20]
Chinese	0.22 [0.13, 0.32]	0.18 [0.07, 0.30]	0.27 [0.13, 0.41]	0.22 [0.13, 0.32]	0.18 [0.07, 0.30]	0.27 [0.14, 0.41]
Other	0.19 [0.16, 0.23]	0.15 [0.11, 0.20]	0.24 [0.18, 0.29]	0.19 [0.16, 0.23]	0.15 [0.11, 0.20]	0.23 [0.18, 0.29]
Ethnic variations in overweight†	27.67 (0.002)	19.67 (0.03)	37.33 (<0.001)	22.05 (0.01)	15.98 (0.10)	27.54 (0.002)

Note: Age- and gender-adjusted predictive margins [95% confidence intervals].

Models (1) adjust for year of data collection and exclude those with missing data on equivalized household income.

Models (2) also adjust for equivalized household income.

Only those with unweighted *N*'s of over 30 are included in table.

* Age and gender-specific cut-off points for children extrapolated from adult body mass index cut-offs of 25 kg m^−2^.

† χ^2^ (*P*-value).

**Table 2:** Ethnic differences in the age- and gender-adjusted probabilities of children aged between 2 and 15 being at or above the International Obesity Task Force thresholds for obesity*, rather than normal or under-weight – *combined data across all years*

	Both genders (1)	Boys (1)	Girls (1)	Both genders (2)	Boys (2)	Girls (2)
White English	0.06 [0.06, 0.06]	0.05 [0.05, 0.06]	0.06 [0.06, 0.07]	0.06 [0.06, 0.06]	0.05 [0.05, 0.06]	0.06 [0.06, 0.07]
White Irish	0.06 [0.04, 0.08]	0.04 [0.02, 0.06]	0.08 [0.05, 0.12]	0.06 [0.04, 0.08]	0.04 [0.02, 0.06]	0.09 [0.05, 0.12]
Other White	0.08 [0.06, 0.10]	0.08 [0.06, 0.11]	0.07 [0.05, 0.10]	0.08 [0.06, 0.10]	0.09 [0.06, 0.12]	0.08 [0.05, 0.11]
Black Caribbean	0.09 [0.07, 0.11]	0.07 [0.05, 0.10]	0.11 [0.08, 0.15]	0.09 [0.07, 0.11]	0.07 [0.04, 0.09]	0.11 [0.08, 0.13]
Black African	0.10 [0.07, 0.12]	0.10 [0.07, 0.13]	0.09 [0.06, 0.12]	0.09 [0.07, 0.11]	0.09 [0.06, 0.12]	0.09 [0.06, 0.11]
Indian	0.06 [0.04, 0.07]	0.07 [0.04, 0.09]	0.05 [0.03, 0.06]	0.06 [0.04, 0.07]	0.07 [0.04, 0.09]	0.05 [0.03, 0.06]
Pakistani	0.08 [0.06, 0.10]	0.09 [0.06, 0.12]	0.08 [0.05, 0.11]	0.08 [0.06, 0.09]	0.08 [0.06, 0.11]	0.07 [0.04, 0.10]
Bangladeshi	0.10 [0.06, 0.14]	0.09 [0.02, 0.15]	0.11 [0.06, 0.16]	0.09 [0.05, 0.13]	0.08 [0.02, 0.14]	0.10 [0.05, 0.14]
Other South Asian	0.06 [0.04, 0.08]	0.06 [0.03, 0.08]	0.06 [0.04, 0.08]	0.06 [0.04, 0.07]	0.06 [0.03, 0.08]	0.06 [0.03, 0.08]
Chinese	0.03 [−0.00, 0.06]	0.03 [−0.02, 0.08]	0.03 [−0.02, 0.08]	0.03 [−0.00, 0.06]	0.03 [−0.02, 0.07]	0.03 [−0.02, 0.08]
Other	0.08 [0.06, 0.11]	0.07 [0.04, 0.10]	0.09 [0.06, 0.13]	0.08 [0.05, 0.10]	0.07 [0.04, 0.10]	0.09 [0.06, 0.12]
Ethnic variations in obesity†	50.27 (<0.001)	19.25 (0.03)	31.14 (<0.001)	29.07 (<0.001)	18.40 (0.04)	26.42 (0.003)

Note: Age- and gender-adjusted predictive margins [95% confidence intervals].

Models (1) adjust for year of data collection and exclude those with missing data on equivalized household income.

Models (2) also adjust for equivalized household income.

Only those with unweighted *N*'s of over 30 are included in table.

* Age and gender-specific cut-off points for children extrapolated from adult body mass index cut-offs of 30 kg/m2.

† χ^2^ (*P*-value).

There were also statistically significant ethnic variations for boys and girls. Black African boys had a higher risk of being obese than white English and white Irish boys. Black African girls were more likely to be overweight than girls in the white English, white Irish and ‘other South Asian’ groups and black Caribbean girls were more likely to be obese compared with girls in the white English and Indian groups. Again, there was an indication that other variations may have become statistically significant with additional sample power.

Adjusting for variations in equivalized household income between the groups had minimal impact on the ethnic variations in overweight/obesity identified, although the ethnic variations in risk of overweight among boys was not statistically significant after adjusting for socioeconomic variation between the groups.

### Analyses using stratified data

Tables 3 and 4 report the results of the analyses stratified by time period for the overweight and obesity, respectively. These analyses allow us to investigate the variation in time trends in overweight and obesity across ethnic groups.

**Table 3:** Ethnic differences in the age- and gender-adjusted probabilities of children aged between 2 and 15 being at or above the International Obesity Task Force thresholds for overweight (but below those for obesity)*, rather than normal or under-weight – *stratified data*

	1998–2001	N	2002–2005	N	2006–2009	N	Variations in overweight over time†
White English	0.18 [0.17, 0.19]	8, 787	0.20 [0.19, 0.21]	10, 554	0.18 [0.18, 0.19]	17, 259	11.96 (0.003)
White Irish	0.16 [0.09, 0.23]	189	0.18 [0.14, 0.23]	505	0.17 [0.12, 0.23]	223	0.25 (0.88)
Other White	0.13 [0.08, 0.19]	195	0.21 [0.15, 0.26]	291	0.19 [0.15, 0.23]	559	3.83 (0.15)
Black Caribbean	0.17 [0.11, 0.22]	645	0.18 [0.14, 0.22]	561	0.19 [0.15, 0.22]	542	0.32 (0.85)
Black African	0.27 [0.18, 0.37]	121	0.28 [0.20, 0.35]	453	0.22 [0.18, 0.26]	544	2.65 (0.27)
Indian	0.21 [0.15, 0.26]	587	0.17 [0.12, 0.22]	582	0.19 [0.15, 0.22]	578	0.82 (0.66)
Pakistani	0.14 [0.09, 0.18]	804	0.21 [0.16, 0.28]	630	0.21 [0.17, 0.25]	657	7.22 (0.03)
Bangladeshi	0.11 [0.05, 0.18]	611	0.20 [0.12, 0.28]	442	0.20 [0.14, 0.27]	221	4.40 (0.11)
Other South Asian	0.15 [0.08, 0.23]	132	0.16 [0.10, 0.21]	180	0.14 [0.11, 0.17]	587	0.60 (0.74)
Chinese	0.05 [−0.04, 0.14]	253	0.16 [0.02, 0.30]	187	0.27 [0.13, 0.41]	49	7.35 (0.03)
Other	0.18 [0.12, 0.24]	269	0.22 [0.16, 0.28]	273	0.20 [0.16, 0.25]	326	0.66 (0.72)
Ethnic variations in overweight†	27.36 (0.002)		10.80 (0.37)		19.04 (0.03)		

Note: Age- and gender-adjusted predictive margins [95% confidence intervals].

Only those with unweighted N's of over 30 are included in table.

* Age and gender-specific cut-off points for children extrapolated from adult body mass index cut-offs of 25 kg m^−2^.

† χ^2^ (*P*-value).

**Table 4:** Ethnic differences in the age- and gender-adjusted probabilities of children aged between 2 and 15 being at or above the International Obesity Task Force thresholds for obesity*, rather than normal or under-weight – *stratified data*

	1998–2001	N	2002–2005	N	2006–2009	N	Variations in obesity over time†
White English	0.05 [0.05, 0.06]	8, 787	0.07 [0.06, 0.07]	10, 554	0.06 [0.06, 0.06]	17, 259	12.65 (0.002)
White Irish	0.02 [0.00, 0.04]	189	0.07 [0.04, 0.10]	505	0.06 [0.03, 0.09]	223	8.53 (0.014)
Other White	0.08 [0.04, 0.11]	195	0.09 [0.05, 0.12]	291	0.07 [0.05, 0.10]	559	0.52 (0.77)
Black Caribbean	0.05 [0.02, 0.08]	645	0.10 [0.07, 0.14]	561	0.11 [0.08, 0.14]	542	10.00 (0.007)
Black African	0.05 [0.01, 0.09]	121	0.11 [0.07, 0.15]	453	0.11 [0.08, 0.13]	544	5.96 (0.051)
Indian	0.05 [0.02, 0.09]	587	0.08 [0.05, 0.11]	582	0.06 [0.04, 0.08]	578	2.08 (0.35)
Pakistani	0.09 [0.04, 0.15]	804	0.08 [0.05, 0.11]	630	0.10 [0.08, 0.12]	657	1.34 (0.51)
Bangladeshi	0.04 [−0.00, 0.07]	611	0.14 [0.08, 0.20]	442	0.12 [0.06, 0.18]	221	9.86 (0.007)
Other South Asian	0.04 [−0.00, 0.08]	132	0.05 [0.02, 0.09]	180	0.06 [0.04, 0.08]	587	1.30 (0.52)
Chinese	−	253	0.02 [0.00, 0.03]	187	0.06 [−0.00, 0.12]	49	8.28 (0.016)
Other	0.05 [0.02, 0.08]	269	0.11 [0.06, 0.16]	273	0.08 [0.05, 0.11]	326	3.85 (0.15)
Ethnic variations in obesity†	463.14 (<0.001)		56.26 (<0.001)		36.19 (<0.001)		

Note: Age- and gender-adjusted predictive margins [95% confidence intervals].

Only those with unweighted N's of over 30 are included in table.

* Age and gender-specific cut-off points for children extrapolated from adult body mass index cut-offs of 30 kg m^−2^.

† χ^2^ (*P*-value).

First, with the exception of variations in overweight in 2002–2005, there were significant ethnic variations in overweight and obesity in each period after adjusting for age and gender differences between the groups, as shown by the Wald tests for the joint significance of the ethnic categories. There was no significant interaction between ethnicity and year of data collection for models predicting either overweight or obesity. The findings for ethnic variations in overweight and obesity for each year separately, shown as supplementary material, followed these broad trends, although they also suggested significant variation in the prevalence of overweight among ‘other white’ and Bangladeshi, and obesity among white Irish, black African and ‘other South Asian’ children over time, which were not identified in the combined (4-year) models.

**Figure 1:**
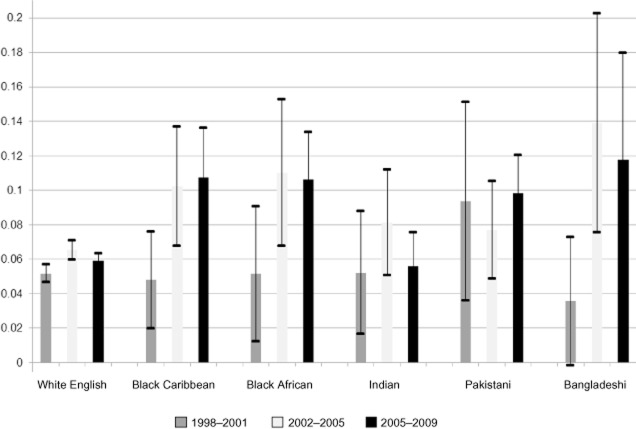
Ethnic differences in the probability (with 95% confidence intervals) of obesity by year of data collection, adjusted for age and gender.

With regards to the time trends, significant variation over time in the prevalence of overweight and obesity can be identified for some, but not all, ethnic categories. The figures suggest that the proportion of white English children at or above the threshold for overweight (but below those for obesity) peaked at 20% in the period 2002–2005, with the proportion defined as overweight in 2006–2009 the same as that in 1998–2001 (Table 3). The proportion at or above the threshold for obesity also appears to have peaked for white English groups during this period (at 7%), although figures for the 2006–2009 period remained somewhat higher than those for 1998–2001 (Table 4). Figure 1 shows the variation in the probability of being obese over time for a selection of ethnic groups using data presented in Table 4. Despite having (generally) higher levels of obesity in the early period, white English groups have consistently lower obesity levels than most other groups in the period since 2002, with less variation over time.

The results in Tables 3 and 4 also suggest that levels of overweight and obesity may have also peaked among other ethnic groups – specifically those for overweight among ‘other white’, white Irish, black African, ‘other South Asian’ and ‘other’ ethnic groups, and obesity among white Irish, ‘other white’, black African, Indian, Bangladeshi and ‘other’ groups. However, there is evidence that the prevalence of overweight and obesity may continue to rise among other ethnic minorities examined. The estimated risk of children being overweight increased over the study period among black Caribbean and Chinese ethnic groups, while obesity rates also continued to rise for children in black Caribbean, Pakistani, ‘other South Asian’ and Chinese groups.

## Discussion

This study has identified ethnic variations in the prevalence of overweight and obesity in England. These variations persisted after controlling for ethnic variations in household income. This is the first study to report detailed analysis of longitudinal trends in child and adolescent overweight and obesity among ethnic groups in England. Perhaps not surprisingly, the white English group followed trends similar to those reported for the whole English population, with obesity prevalence peaking in the mid-2000s and some evidence of decline thereafter. We identified no clear or generalizable trends in the prevalence of overweight or obesity among the other ethnic groups examined. However, it appears unlikely that overweight and obesity are in decline among all these groups. Overall, there were higher risks of both overweight and obesity among black African and obesity among black Caribbean children, although these were not consistently replicated in separate gender models. There is evidence to suggest that these levels may have peaked among black African children, but not among black Caribbean.

An important conclusion to draw from this work relates to the complexity of the relationships between ethnicity and BMI over time, although the variations in the sampling methodologies adopted and sample power available in the different years makes a clearer identification of trends problematic. The higher risk of black African children and young people being at or above the threshold for overweight and obesity concurs with some 6,9, but not all 7, earlier research. However, much of this work analyses broader ethnic categories than those used here. Our findings establish the value offered by analyses, which are able to distinguish between different black, Asian and white groups. It also adds further detail to our understanding of the complex relationships between ethnicity and BMI.

Defining overweight and obesity among young people is problematic. BMI is an indirect measure of body fat, but it remains the best single measure to use in large-scale epidemiological studies as it is more acceptable and reproducible than skin-fold measures 30. It has been argued that other measures of overweight and obesity produce indicators, which can more usefully relate to central adiposity in adults and may identify ethnic variations in obesity/overweight, which are not identified by measures such as BMI. However, the extent to which such markers are valuable for use with children, as well as the cut-offs to use to identify overweight/obesity, have not been established as comprehensively as they have for adults 31. The international reference curves used here have been debated 6,7,24,32,33, but also widely adopted and are valuable because they provide prevalence rates that are directly comparable to the corresponding prevalence rates in adults. They are also considered the most appropriate for use in the UK, including with multi-ethnic samples 34.

We have not taken account of the potential correlation between BMI and height, and this may overestimate the proportions of overweight in taller groups, such as black Caribbean boys and black African girls, and underestimate that in shorter groups, such as Chinese boys and Bangladeshi girls 35. Adjusting for height differences explained the higher risk of overweight and obesity among black African and Caribbean groups in the National Child Measurement Programme, but not the higher risk of overweight and obesity among Bangladeshi and Pakistani nor the lower risk of Chinese groups compared with white British children 36.

There are other factors that may drive ethnic inequalities in overweight/obesity or health more generally among adults and children, which have not been considered here. For example, studies in both the UK and US have repeatedly identified an ethnic variation in body shape perception. Several 37–39, although not all 40, studies have found African American and black British young people to hold more positive perceptions of larger body sizes, with those with a higher BMI in these groups more frequently identifying themselves as ‘normal weight’ than those with other ethnicities. While the greater body satisfaction expressed by these black children may protect them from developing particular unhealthy food habits; that it remains at larger sizes might also indicate that the negative health implications of having larger BMIs are not so well appreciated in this group. Such findings may suggest an opportunity for education in this area.

There are also some, but not conclusive, empirical evidence for a positive relationship between experience of forms of racist victimization and higher BMI among adults 41, although research has not examined this association in children 42. Given the strength of the impact of racist victimization on the health of minoritized groups identified by these reviews, further investigation of these effects may be warranted.

In conclusion, this paper provides clear evidence that rates of overweight/obesity among children and young people vary by ethnicity over time, with rates of decline seen among certain white groups not apparent among those with other ethnicities. We have also established that while certain black groups have a particularly high prevalence of overweight and obesity, these patterns, and how they may change in the future, vary between them. The association between ethnicity and BMI was not explained by socioeconomic differences between the groups; a finding consistent with others from the UK 6,7, but not all those produced elsewhere 43. These findings remain somewhat surprising, particularly given the significant role of structural effects in the generation of ethnic health inequalities more generally 29. While such findings may implicate certain lifestyle factors, the (lack of) ethnic variation in markers of physical activity and fruit and vegetable consumption identified in these data 35 would not seem to justify this assumption. Moreover, such approaches do not appreciate the potential interrelationships between socioeconomic and lifestyles factors in their impact on BMI 43. It therefore seems likely that these variations are driven by differences in the effects of lifestyle mechanisms rather than differences in lifestyles themselves.

Identifying whether, and understanding why, the impact of particular lifestyle and other factors might vary between children with different ethnicities, and therefore how to respond to these, is beyond the scope of this work. But we would suggest that an effective response could be the development of culturally appropriate educational policies, which encourage healthy lifestyles among black and minority ethnicity young people, which can engage with the additional complexities associated with these choices during childhood and particularly adolescence 44,45.

## Supporting information

**Table S1.** Ethnic differences in the age- and gender-adjusted probabilities of children aged between 2 and 15 being at or above the IOTF thresholds for overweight (but below those for obesity) and obesity^1^, rather than normal or under-weight, by year of data collection.
