# Supplementary material for: Ethnic variations in overweight and obesity among children over time: findings from analyses of the Health Surveys for England 1998–2009
Source: Pediatr Obes. 2013 Apr 2;9(3):186–96. doi: 10.1111/j.2047-6310.2013.00159.x (PMC4171811; doi:10.1111/j.2047-6310.2013.00159.x)
Supplement: Supplementary file 1 — Table S1. Ethnic differences in the age- and gender-adjusted probabilities of children aged between 2 and 15 being at or above the IOTF thresholds for overweight (but below those for obesity) and obesity1, rather than normal or under-weight, by year of data collection. [file ijpo0009-0186-SD1.doc]

Supplementary material: Ethnic differences in the age- and gender-adjusted probabilities of children aged between 2 and 15 being at or above the IOTF thresholds for overweight (but below those for obesity) and obesity^1^, rather than normal or under-weight, by year of data collection.

Age- and gender-adjusted predictive margins (95% confidence intervals).

|  |  | White English | White Irish | Other White | Black Caribbean | Black African | Indian | Pakistani | Bangladeshi | Other South Asian | Chinese | Other | Ethnic variations in overweight^2^ |
| --- | --- | --- | --- | --- | --- | --- | --- | --- | --- | --- | --- | --- | --- |
| 1998/1999 | Overweight | 0.18 (0.17,0.19) | 0.22 (0.09,0.35) | 0.22 (0.07,0.36) | 0.16 (0.09,0.22) | 0.29 (0.16,0.41) | 0.12 (0.08,0.17) | 0.12 (0.07,0.17) | 0.13 (0.03,0.23) | 0.14 (0.01,0.26) | 0.06 (-0.04,0.16) | 0.17 (0.09,0.25) | 21.92(0.015) |
|  | Obese | 0.05 (0.04,0.06) | 0.09 (0.01,0.17) | 0.06 (-0.18,0.14) | 0.07 (0.02,0.11) | 0.04 (-0.01,0.09) | 0.02 (0.00,0.04) | 0.07 (-0.00,0.15) | 0.01 (0.00,0.02) | - | - | 0.06 (0.02,0.10) | 247.38(<0.001) |
| 2000 | Overweight | 0.17 (0.15,0.20) | 0.10 (-0.09,0.29) | 0.11 (0.02,0.21) | 0.14 (0.03,0.25) | 0.20 (-0.02,0.42) | 0.31 (0.15,0.47) | 0.09 (-0.03,0.21) | 0.09 (-0.02,0.20) | 0.08 (-0.02,0.19) | - | 0.16 (0.03,0.28) | 309.60(<0.001) |
|  | Obese | 0.06 (0.04,0.07) | - | 0.06 (0.01,0.12) | 0.02 (-0.02,0.07) | - | 0.11 (0.00,0.23) | 0.08 (-0.03,0.18) | 0.10 (-0.00,0.20) | - | - | 0.07 (-0.02,0.17) | 85.14(<0.001) |
| 2001 | Overweight | 0.20 (0.18,0.21) | 0.16 (0.07,0.25) | 0.11 (0.04,0.18) | 0.20 (0.09,0.31) | 0.33 (0.17,0.48) | 0.31 (0.20,0.42) | 0.19 (0.09,0.28) | 0.08 (-0.03,0.20) | 0.19 (0.07,0.32) | - | 0.22 (0.10,0.34) | 617.12(<0.001) |
|  | Obese | 0.05 (0.04,0.06) | - | 0.09 (0.03,0.16) | 0.04 (-0.01,0.09) | 0.13 (0.02,0.25) | 0.05 (-0.01,0.11) | 0.13 (0.02,0.24) | - | 0.07 (-0.01,0.14) | - | 0.03 (-0.01,0.07) | 143.63(<0.001) |
| 2002 | Overweight | 0.20 (0.18,0.21) | 0.23 (0.14,0.32) | 0.17 (0.11,0.23) | 0.17 (0.10,0.24) | 0.31 (0.18,0.43) | 0.13 (0.08,0.18) | 0.18 (0.09,0.26) | 0.20 (0.08,0.32) | 0.15 (0.08,0.22) | 0.20 (-0.14,0.53) | 0.19 (0.11,0.27) | 10.78(0.38) |
|  | Obese | 0.06 (0.06,0.07) | 0.07 (0.03,0.12) | 0.08 (0.04,0.12) | 0.08 (0.03,0.14) | 0.08 (0.02,0.15) | 0.08 (0.03,0.13) | 0.08 (0.03,0.13) | 0.13 (0.02,0.24) | 0.05 (0.01,0.09) | - | 0.10 (0.03,0.17) | 402.77(<0.001) |
| 2003/2004 | Overweight | 0.21 (0.19,0.22) | 0.17 (0.12,0.22) | 0.26 (0.13,0.38) | 0.18 (0.12,0.24) | 0.26 (0.16,0.35) | 0.19 (0.11,0.27) | 0.25 (0.17,0.33) | 0.28 (0.14,0.42) | 0.18 (0.05,0.30) | 0.17 (0.10,0.24) | 0.27 (0.17,0.38) | 9.12(0.52) |
|  | Obese | 0.07 (0.06,0.08) | 0.06 (0.03,0.09) | 0.09 (0.03,0.15) | 0.09 (0.05,0.13) | 0.14 (0.08,0.21) | 0.07 (0.03,0.11) | 0.05 (0.02,0.08) | 0.17 (0.05,0.28) | 0.08 (-0.01,0.17) | 0.03 (0.01,0.06) | 0.12 (0.04,0.20) | 16.23(0.093) |
| 2005 | Overweight | 0.21 (0.19,0.23) | 0.19 (0.05,0.33) | 0.31 (0.09,0.53) | 0.21 (0.09,0.32) | 0.27 (0.10,0.45) | 0.20 (0.02,0.37) | 0.23 (0.06,0.39) | 0.04 (-0.02,0.10) | 0.14 (0.04,0.25) | - | 0.08 (-0.01,0.18) | 389.11(<0.001) |
|  | Obese | 0.07 (0.05,0.08) | 0.19 (0.03,0.34) | 0.14 (0.02,0.25) | 0.22 (0.12,0.34) | 0.12 (0.02,0.23) | 0.12 (0.02,0.22) | 0.15 (0.04,0.26) | 0.10 (0.02,0.18) | 0.02 (-0.02,0.05) | - | 0.03 (-0.03,0.10) | 150.95(<0.001) |
| 2006 | Overweight | 0.19 (0.17,0.20) | 0.12 (0.05,0.18) | 0.21 (0.14,0.28) | 0.18 (0.12,0.23) | 0.26 (0.19,0.33) | 0.17 (0.10,0.24) | 0.22 (0.16,0.29) | 0.24 (0.11,0.36) | 0.17 (0.09,0.25) | 0.22 (-0.06,0.50) | 0.21 (0.12,0.29) | 11.75(0.30) |
|  | Obese | 0.06 (0.05,0.07) | 0.02 (-0.01,0.05) | 0.05 (0.01,0.09) | 0.11 (0.06,0.16) | 0.09 (0.03,0.14) | 0.05 (0.01,0.09) | 0.10 (0.06,0.14) | 0.10 (-0.04,0.24) | 0.05 (0.01,0.09) | - | 0.10 (0.04,0.16) | 307.67(<0.001) |
| 2007 | Overweight | 0.19 (0.17,0.20) | 0.24 (0.13,0.35) | 0.25 (0.17,0.33) | 0.21 (0.15,0.28) | 0.17 (0.11,0.24) | 0.19 (0.13,0.25) | 0.21 (0.14,0.28) | 0.24 (0.11,0.37) | 0.09 (0.04,0.14) | 0.56 (0.25,0.87) | 0.21 (0.14,0.29) | 26.61(0.003) |
|  | Obese | 0.06 (0.05,0.07) | 0.08 (0.03,0.13) | 0.09 (0.04,0.13) | 0.09 (0.04,0.15) | 0.08 (0.03,0.12) | 0.09 (0.04,0.13) | 0.10 (0.05,0.15) | 0.14 (0.03,0.26) | 0.03 (-0.00,0.07) | 0.07 (-0.07,0.21) | 0.05 (0.01,0.09) | 12.83(0.23) |
| 2008 | Overweight | 0.18 (0.17,0.19) | 0.23 (0.03,0.43) | 0.12 (0.06,0.17) | 0.17 (0.10,0.24) | 0.23 (0.15,0.30) | 0.20 (0.14,0.26) | 0.22 (0.14,0.29) | 0.15 (0.06,0.25) | 0.16 (0.11,0.21) | 0.11 (-0.03,0.26) | 0.13 (0.05,0.21) | 12.20(0.27) |
|  | Obese | 0.06 (0.05,0.07) | 0.05 (-0.04,0.15) | 0.08 (0.03,0.13) | 0.11 (0.06,0.17) | 0.13 (0.08,0.18) | 0.04 (0.01,0.06) | 0.10 (0.06,0.14) | 0.14 (0.04,0.24) | 0.07 (0.04,0.11) | - | 0.05 (-0.01,0.11) | 374.10(<0.001) |
| 2009 | Overweight | 0.18 (0.16,0.19) | - | 0.18 (0.08,0.28) | 0.20 (0.11,0.29) | 0.24 (0.15,0.33) | 0.19 (0.09,0.30) | 0.18 (0.08,0.28) | 0.17 (0.01,0.33) | 0.11 (0.05,0.17) | 0.25 (-0.01,0.51) | 0.31 (0.10,0.51) | 528.25(<0.001) |
|  | Obese | 0.06 (0.05,0.07) | 0.18 (0.00,0.37) | 0.07 (0.01,0.12) | 0.13 (0.05,0.21) | 0.14 (0.06,0.22) | 0.04 (0.00,0.08) | 0.09 (0.04,0.14) | 0.07 (0.00,0.14) | 0.08 (0.03,0.14) | 0.23 (0.02,0.45) | 0.15 (0.03,0.27) | 17.08(0.073) |
|  |  |  |  |  |  |  |  |  |  |  |  |  |  |
| Variations in | Overweight | 17.43(0.04) | 133.22(<0.001) | 17.63(0.04) | 2.75(0.97) | 7.75(0.56) | 15.13(0.087) | 13.81(0.13) | 20.69(0.014) | 7.25(0.61) | 45.52(<0.001) | 10.74(0.29) |  |
| Overweight/ obesity over time^2^ | Obese | 14.23(0.11) | 48.10(<0.001) | 3.87(0.92) | 20.68(0.014) | 94.56(<0.001) | 15.78(0.072) | 7.94(0.54) | 49.41(<0.001) | 50.25(<0.001) | 10.90(0.012) | 10.52(0.31) |  |

^1^Age and gender specific cut-off points for children extrapolated from adult BMI cut-offs of 25kg/m^2^ (for overweight) and 30kg/m^2^ (for obesity)

^2^ Chi^2^ (p-value)
